# 

**Published:** 1860-12

**Authors:** 


### BOUND VOLUMES.

The Journal of Health for eighteen hundred and sixty, is already bound uniformly with the previous volumes, with a copious index and title-page. We will exchange it at our office for the loose numbers, and twenty-five cents in addition to pay for the binding. Missing numbers will be supplied at eight cents each.

The old numbers of the Journal, previous to eighteen hundred and sixty, will be received at our office until February next, at five cents each, for new subscriptions on any of our publications. We can print them for two cents each, but the offer is made to encourage our subscribers to have the Journal of Health in the more substantial form of a bound volume, for the character of the articles is such, that they will be almost if not quite, as practical and useful in nineteen hundred as they are now.

